# Distinct routes to metastasis: plasticity-dependent and plasticity-independent pathways

**DOI:** 10.1038/onc.2015.497

**Published:** 2016-01-11

**Authors:** J A Somarelli, D Schaeffer, M S Marengo, T Bepler, D Rouse, K E Ware, A J Hish, Y Zhao, A F Buckley, J I Epstein, A J Armstrong, D M Virshup, M A Garcia-Blanco

**Affiliations:** 1 Center for RNA Biology, Duke University Medical Center, Durham, NC, USA; 2 Department of Molecular Genetics and Microbiology, Duke University Medical Center, Durham, NC, USA; 3 Department of Laboratory Animal Resources, Duke University Medical Center, Durham, NC, USA; 4 Department of Medicine, Duke Cancer Institute, Duke University Medical Center, Durham, NC, USA; 5 Department of Radiation Oncology, Duke University Medical Center, Durham, NC, USA; 6 Department of Pathology, Duke University Medical Center, Durham, NC, USA; 7 Department of Pathology, Johns Hopkins Hospital, Baltimore, MD, USA; 8 Department of Urology, Johns Hopkins Hospital, Baltimore, MD, USA; 9 Department of Oncology, Johns Hopkins Hospital, Baltimore, MD, USA; 10 Solid Tumor Program and the Duke Prostate Center, Duke Cancer Institute, Duke University Medical Center, Durham, NC, USA; 11 Program in Cancer and Stem Cell Biology, Duke-NUS Graduate Medical School, Singapore, Singapore; 12 Program in Molecular Genetics and Genomics, Duke Cancer Institute, Duke University Medical Center, Durham, NC, USA; 13 Department of Biochemistry and Molecular Biology, University of Texas Medical Branch, Galveston, TX, USA

## Abstract

The cascade that culminates in macrometastases is thought to be mediated by phenotypic plasticity, including epithelial–mesenchymal and mesenchymal–epithelial transitions (EMT and MET). Although there is substantial support for the role of EMT in driving cancer cell invasion and dissemination, much less is known about the importance of MET in the later steps of metastatic colonization. We created novel reporters, which integrate transcriptional and post-transcriptional regulation, to test whether MET is required for metastasis in multiple *in vivo* cancer models. In a model of carcinosarcoma, metastasis occurred via an MET-dependent pathway; however, in two prostate carcinoma models, metastatic colonization was MET independent. Our results provide evidence for both MET-dependent and MET-independent metastatic pathways.

## Introduction

Epithelial plasticity, including epithelial–mesenchymal transitions (EMT) and the reverse, mesenchymal–epithelial transitions (MET), is thought to mediate different stages of the metastatic cascade. EMT is postulated to confer upon cancer cells the ability to detach from the primary tumor, degrade the basement membrane and invade locally or distally.^1, 2^ Upon seeding, metastatic post-EMT cells are hypothesized to revert back to an epithelial state via MET.^1, 2, 3^ For example, E-cadherin, a widely used epithelial-specific biomarker, is observed in clinical metastases from breast, prostate and pancreatic cancers.^4, 5, 6, 7, 8^ In addition, Ocaña *et al.*^9^ showed that downregulation of the EMT-inducing transcription factors PRRX1 and TWIST1 was necessary for metastasis to the lungs.^9^ Similarly, in a squamous cell carcinoma model, Tsai *et al.*^10^ observed that downregulation of TWIST1 subsequent to metastatic dissemination led to significantly more lung macrometastases.^10^ Furthermore, Stankic *et al.*^11^ showed that the transcription factor inhibitory protein, ID1, enhances metastatic colonization by inhibition of TWIST1.^11^ Yet, despite these findings, other studies suggest that MET may have an inhibitory effect on metastasis.^12, 13, 14, 15^ Indeed, the current literature suggests that the importance of MET in metastasis may be cell type and/or context dependent.

Our group has generated fluorescence-based reporters of epithelial plasticity to visualize phenotypic transitions *in vivo*.^16, 17, 18^ We reasoned that our MET reporters could be useful to test the hypothesis that MET is a modulator of metastatic colonization. Along these lines, we developed a lineage-tracing reporter based on combined transcription and alternative splicing regulatory elements to measure the frequency of MET-like events during tumor growth and metastasis. Using the lineage-tracing reporter system, we were able to quantify, for the first time, overall frequencies of MET during primary tumor and metastatic growth. Remarkably, we observed that the frequency of MET within primary tumors and metastatic nodules was not significantly different, with very low rates of MET taking place during the growth of tumors and metastases. In a parallel series of experiments, we also created a suicide reporter that kills cells undergoing MET to test the hypothesis that MET is required for metastatic colonization in multiple models of metastasis. Surprisingly, the suicide reporters revealed the existence of both MET-dependent and MET-independent metastatic cascades.

## Results

### Examining MET biomarkers in human clinical samples

Numerous investigations have illuminated the important role of EMT in driving metastatic dissemination; however, our understanding of the need for MET in later steps of the metastatic cascade is limited. To address this question, we started by attempting to gain a better understanding of the spectrum of epithelial-like and mesenchymal-like phenotypes within clinical metastatic specimens. To this end, we interrogated publically-available gene expression data from human laser capture microdissected primary prostate tumors and bone metastases.^19^ Subsequent to dissection, these samples were further evaluated pathologically to ensure that both the primary tumor and metastatic samples contained a large majority of tumor cells.^19^ This data set was selected because it was processed in such a way as to minimize contamination from healthy or tumor-associated stroma. Whole genome expression analysis from *n*=22 primary tumors and *n*=33 bone marrow metastases from patients with metastatic, castration-resistant prostate cancer^19^ showed significant downregulation of epithelial biomarkers and significant upregulation of mesenchymal markers in metastatic samples compared with primary tumors (GSE32269; Supplementary Figure S1). The relative increase in mesenchymal biomarkers in metastases is consistent with the importance of EMT for the metastatic cascade, but does not shed light on whether a transient MET was required for metastatic colonization. We therefore decided to examine a role for MET in the metastatic cascade.

### Post-EMT AT3 cells undergo MET during tumor formation

**Figure 1:**
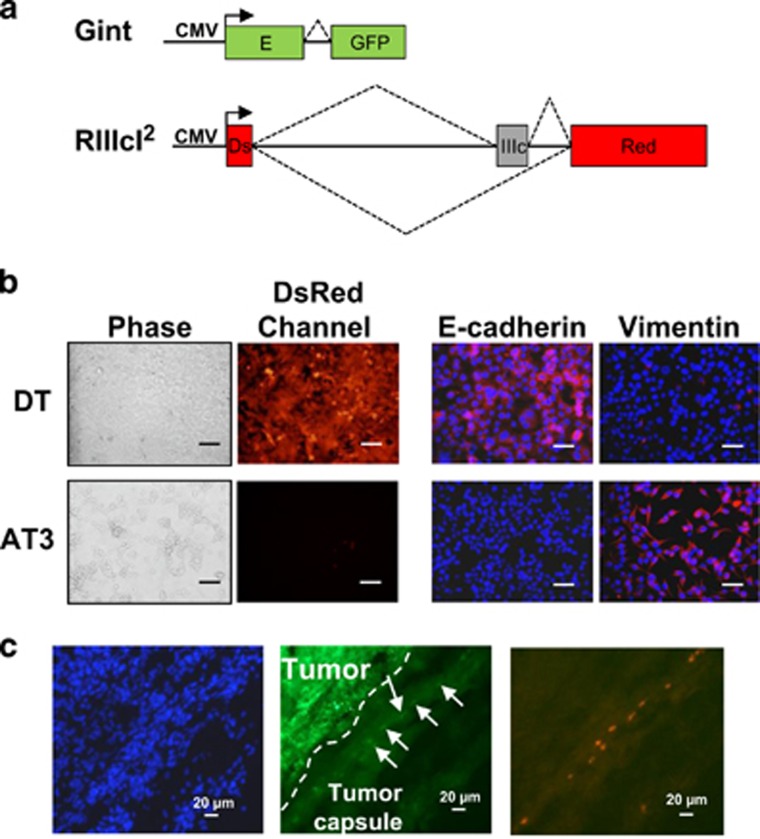
Fluorescence-based MET reporters reveal epithelial plasticity in mesenchymal rat prostate tumors. (**a**) Schematic of the Gint and RIIIcI^2^ reporters. (**b**) Skipping of exon IIIc leads to production of DsRed in epithelial DT cells, while inclusion of exon IIIc in mesenchymal AT3 cells prevents the expression of DsRed. Epithelial DT cells express high levels of E-cadherin with low vimentin, while mesenchymal AT3 cells have almost no E-cadherin, but robustly express vimentin. (**c**) AT3+Gint+RIIIcI^2^ tumors reveal foci of DsRed-expressing cells near the tumor edge. A dotted line indicates the border between the inner tumor mass and the unlabeled cells of the tumor capsule. Arrows highlight tumor cells that reside within the tumor capsule that have undergone MET. Scale bars=50 μm unless otherwise specified.

To monitor MET *in vivo*, we developed a fluorescence-based reporter of MET, which was driven by epithelial-specific skipping of exon IIIc from fibroblast growth factor receptor 2 (FGFR2) (Figure 1a).^16, 17, 18, 20^ Epithelial cells skip exon IIIc, while mesenchymal cells include exon IIIc. The RIIIcI^2^ reporter contains the DsRed open reading frame (ORF) interrupted by the IIIc exon and flanking introns.^20^ Skipping of exon IIIc in epithelial Dunning rat prostate cancer DT cells leads to an in-frame DsRed mRNA and expression of DsRed (Figure 1b), while exon IIIc inclusion in mesenchymal AT3 cells interrupts the DsRed ORF, with little to no DsRed expression (Figure 1b). DsRed expression correlated with high E-cadherin staining and low levels of vimentin in the epithelial DT cells. Conversely, the mesenchymal AT3 cells expressed little to no DsRed, low E-cadherin and high levels of vimentin (Figure 1b).

Using this reporter, our group observed MET-like events within AT3 mesenchymal rat prostate tumors and metastases;^16^ however, these studies were limited by the lack of a tumor-specific label as a denominator to determine the amount of MET within the primary tumors. To quantify the number of cells that undergo MET within AT3 primary tumors, we engineered AT3 cells harboring RIIIcI^2^ and Gint, the latter of which contains the enhanced green fluorescent protein (EGFP) ORF interrupted by a constitutively spliced intron. The Gint reporter allows labeling of all tumor cells, while RIIIcI^2^ reports on cellular phenotype (epithelial or mesenchymal) at a specific moment in time. As previously reported,^16^ we observed rare foci of MET within AT3 primary tumors (Figure 1c). Using flow cytometry (*n*=6), we calculated the prevalence of MET at a specific time during tumor growth. We counted an average of 91 489 EGFP+ events per tumor and estimated that an average of 262 of these EGFP+ cells also express DsRed, indicating that MET is a rare event (0.28±0.04%) within AT3 primary tumors.

### Combinatorial control enables fine tuning of reporter expression

Although the RIIIcI^2^ reporter provides unique information about the proportion of cells undergoing MET at a specific moment in time, we postulated that transitions between epithelial and mesenchymal states among highly aggressive cancer cells could be highly reversible, and we sought to quantify the overall transition frequency during tumor progression (the incidence rate of MET). To accomplish this, we developed a lineage-tracing reporter based on Cre recombinase. Considering that extremely low levels of Cre recombinase can trigger removal of genetic elements flanked by *LoxP* sites, we hypothesized that the alternative splicing-based reporters may require additional control elements to enhance specificity of expression. As hoped, the combinatorial use of promoter and splicing elements in E-cadFFIIIcI^2^ had a multiplicative effect, providing over 50-fold higher expression of Firefly luciferase in epithelial DT cells compared with mesenchymal AT3 cells (Supplementary Figure S2). Perhaps more importantly, the expression of Firefly luciferase in mesenchymal AT3 cells was very low, indeed barely above background. These assays validated the combinatorial use of transcriptional and post-transcriptional control elements to provide remarkable specificity among cell types and formed the basis for the use of highly sensitive enzymatic reactions as reporters of cell fate and phenotype.

### Lineage tracing reporters to track MET *in vivo*

**Figure 2:**
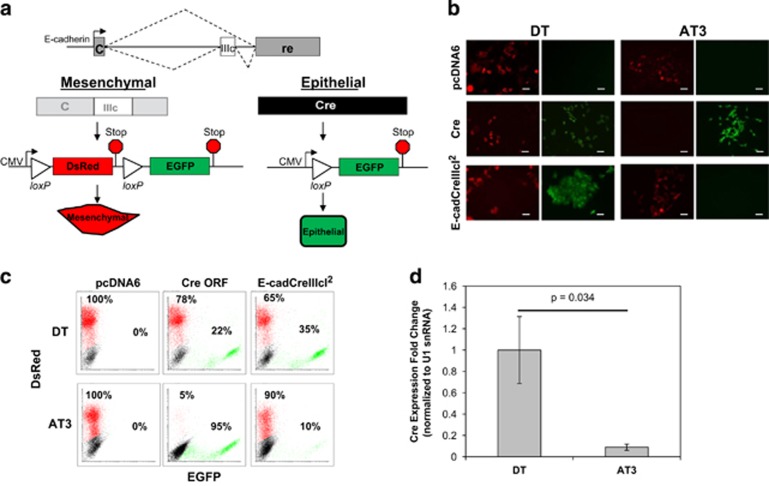
Design and validation of lineage-tracing reporters of MET. (**a**) Conceptual design of E-cadCreIIIcI^2^. In mesenchymal cells, the E-cadherin promoter is inactive and the IIIc exon is included; no Cre is produced. In epithelial cells, Cre is actively transcribed from the E-cadherin promoter and exon IIIc is efficiently skipped, leading to expression of Cre recombinase. E-cadCreIIIcI^2^ acts on RG, which contains a DsRed ORF and stop codon (red octagons) flanked by *Loxp* sites (triangles) followed by the EGFP ORF and a stop codon. MET leads to activation of Cre recombinase and a switch from DsRed to EGFP expression. (**b**) DT and AT3 cells were stably transfected with RG. Cells subsequently transfected with pcDNA6 exclusively expressed DsRed, and cells harboring the Cre ORF activated EGFP expression. When transfected with the E-cadCreIIIcI^2^ reporter, only epithelial DT cells switched from DsRed to EGFP expression, while AT3 cells maintained DsRed expression. Scale bar=50 μm. (**c**) Flow-cytometry analysis shows that a small sub-population of AT3 cells undergo MET. (**d**) RT-qPCR of DT and AT3 cells transfected with RG and E-cadCreIIIcI^2^ reveals approximately 10-fold higher expression of Cre in epithelial DT cells than in mesenchymal AT3 cells.

Validation of the combinatorial reporters indicated that multiple regulatory elements provided enhanced specificity, which is critical for faithful reporter readout. As a result, the lineage-tracing MET reporters were generated based on the design of E-cadFFIIIcI^2^, in which the Firefly ORF was replaced by the Cre recombinase ORF to give E-cadCreIIIcI^2^. Low E-cadherin promoter activation and exon IIIc inclusion in mesenchymal cells should lead to very low, if any, Cre expression (Figure 2a). Conversely, epithelial cells should express Cre via activation of the E-cadherin promoter and exon IIIc skipping (Figure 2a). The E-cadCreIIIcI^2^ reporter acts on a second plasmid, Red/Green (RG),^21^ in which the DsRed ORF containing a stop codon is flanked by *LoxP* sites followed by the EGFP ORF containing a stop codon (Figure 2a). Expression of Cre during MET should lead to permanent removal of DsRed by recombination at *LoxP* sites and consistent expression of EGFP.

To test whether or not the lineage-tracing reporters would accurately reflect cellular phenotype, DT and AT3 cells harboring RG were stably transfected with either an empty vector control, a plasmid that constitutively expressed Cre recombinase, or E-cadCreIIIcI^2^. We have used these cells as models of pre- and post-EMT prostate carcinoma, and have noted that AT3 metastases display markers of MET.^9^ As expected, cells transfected with pcDNA6 remained DsRed+, while both DT and AT3 cells expressing the Cre ORF contained EGFP+ cells (Figure 2b). Interestingly, we observed a subset of DT cells that remained DsRed+ after transfection with both a constitutively expressing Cre ORF and E-cadCreIIIcI^2^ (Figure 2b). The presence of DsRed+ cells in the DTs transfected with the Cre ORF positive control suggested that these DT cells were not properly integrating the reporters. Indeed, PCR for Cre DNA in sorted populations of DsRed+ and EGFP+ DT and AT3 cells revealed that Cre was largely missing from the Dsred+ DT cells, but was strongly positive in the other cell populations (Supplementary Figure S3A). To rule out that the DsRed+ DT cells had undergone a transition and were now mesenchymal, we performed qPCR for the EMT transcription factors and master regulators Zeb1, Zeb2, Snail, Slug and Twist. Only Zeb1 and Slug were detectable in the DT cells; however, there was no difference in Zeb1 and Slug in the DsRed+ and EGFP+ populations (Supplementary Figure S3B). Importantly, only the epithelial DT cells expressed EGFP when transfected with E-cadCreIIIcI^2^ whereas the mesenchymal AT3 cells transfected with E-cadCreIIIcI^2^ were largely EGFP− (Figures 2b and c) despite having high levels of Cre DNA (Supplementary Figure S3A). Flow-cytometric analysis confirmed the results observed by microscopy, except that the more sensitive flow cytometry identified a small sub-population (~10%) of EGFP+ AT3 cells (Figure 2c). Analysis of transcript abundance by qRT-PCR revealed that DT cells containing E-cadCreIIIcI^2^ expressed nearly 10-fold more Cre mRNA than AT3 cells with E-cadCreIIIcI^2^ (Figure 2d). Taken together, these results indicate that the lineage-tracing reporters accurately reflected phenotypic status of epithelial and mesenchymal cells.

### Quantifying MET frequency during tumor growth and metastasis

**Figure 3:**
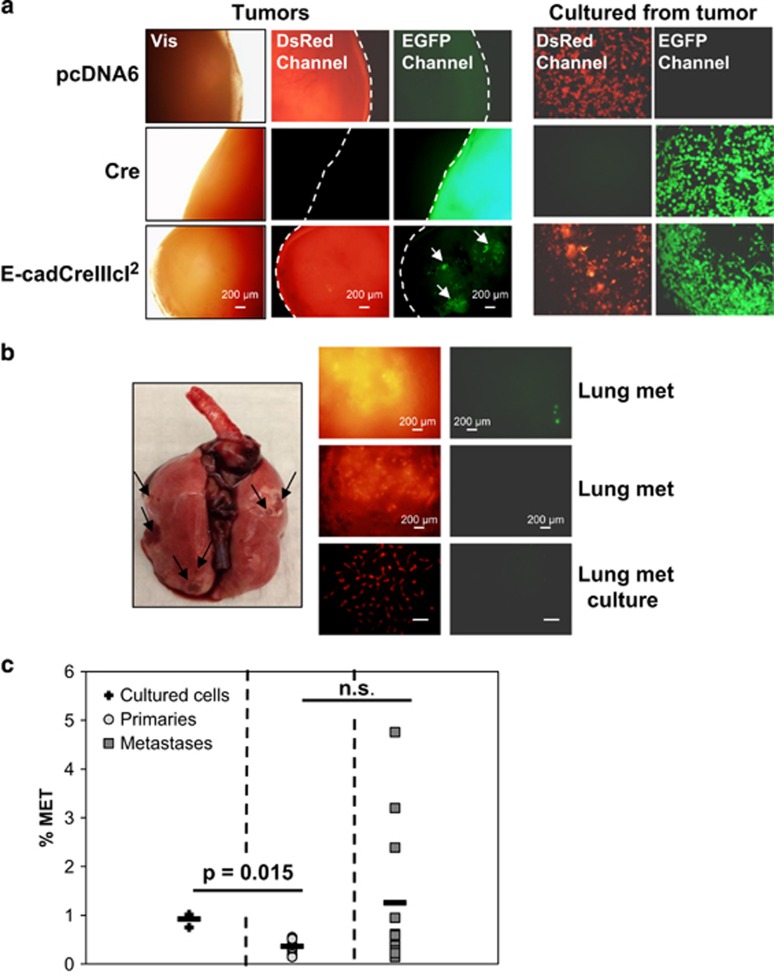
Lineage tracing suggests that MET is rare during tumorigenesis and metastasis. (**a**) Tumors from AT3 cells harboring RG and either pcDNA6, constitutively active Cre, or E-cadCreIIIcI^2^ express DsRed, EGFP and a mixture of both fluorescence proteins (white arrows), respectively. Dotted white lines highlight the outer edges of the tumors. Cultured cells from each tumor type are indicated in the right set of panels. A mixture of DsRed-positive and EGFP-positive cells were cultured from tumors harboring the MET reporter, E-cadCreIIIcI^2^. (**b**) A representative, whole mount image of the lungs, with visible metastases indicated with black arrows. AT3 metastases maintained DsRed expression, with only rare MET-like events, suggesting an MET-independent route to metastasis. (**c**) The percent MET in cultured cells (+ symbol), primary tumors (○ symbol) and lung metastases (□ symbol) was quantified by flow cytometry.

The data above suggested that the E-cadCreIIIcI^2^ reporter could be used to determine the frequency of MET events in tumors. AT3 cells stably transfected with RG and E-cadCreIIIcI^2^ were injected subcutaneously (s.c.) into the left flanks of male Copenhagen or nude rats. As observed with AT3 cells in culture, primary tumors from cells transfected with pcDNA6 or Cre ORF reporters exclusively expressed DsRed or EGFP, respectively (Figure 3a). Interestingly, tumors from cells harboring RG and E-cadCreIIIcI^2^ were predominantly DsRed+, but contained multiple foci of EGFP+ cells (Figure 3a; white arrows).

Cells cultured from tumor explants maintained their fluorescence expression profiles for over 10 days of culture (Figure 3a); cells from tumors containing pcDNA6 or Cre were DsRed+ and EGFP+, respectively, while both DsRed+ and EGFP+ cells were observed from tumors containing E-cadCreIIIcI^2^ (Figure 3a). The morphology of the EGFP+ cells cultured from E-cadCreIIIcI^2^ tumor explants appeared to be mesenchymal, which prompted us to investigate whether these cells, which had undergone MET at some point during tumor growth, had transitioned back to a mesenchymal phenotype. Indeed, single EGFP+ cells exhibited a mesenchymal pattern of biomarker expression, with low E-cadherin and high N-cadherin and fibronectin (Supplementary Figure S4A). This suggests that the MET-like event that led to activation of Cre and a switch from DsRed to EGFP was transient, and that these cells had either transitioned back to a mesenchymal phenotype or had only activated an epithelial-like program long enough to express Cre, but not alter cellular morphology and robustly express E-cadherin.

We also hypothesized that if MET was essential for metastatic colonization of the lungs during the later stages of the metastatic cascade, then the number of cells that underwent an MET-like event would be significantly higher in the lungs than in the post-EMT-like primary tumors. The tail vein (t.v.) metastasis model, in which metastatic cells are injected directly into the circulation to seed and potentially colonize the lungs, was specifically chosen to interrogate the later steps of metastatic colonization. AT3 cells with RG and E-cadCreIIIcI^2^ were injected via the t.v., and lungs were harvested after eight days. To our surprise, we observed EGFP+ AT3 cells only rarely among lung macrometastases (median=1.25% EGFP+ cells; Figures 3b and c), suggesting that the large majority of these metastases formed from cells that had never undergone MET. AT3 cells cultured from lung explants were also predominantly DsRed+ (Figure 3b), with only one EGFP+ colony growing out from the lung explants during the course of these experiments (not shown).

We next sought to quantify by flow cytometry the frequencies of MET within cultured cells (*n*=3), primary tumors (*n*=14) and metastases (*n*=11) to determine whether the number of cells undergoing MET was higher in metastases than in primary tumors. Interestingly, the percentage of MET-like events in primary tumors and lung macrometastases was similar (Figure 3c), with over 95% of cells having never undergone MET during metastatic colonization in the lungs. These data indicated that the overall frequency of MET in AT3 cells was not higher among those that became resident in the lungs.

To verify that lung metastases were indeed mesenchymal, we labeled AT3 cells with Gint and performed t.v. injections. Cryosections from AT3 lung metastases labeled with Gint were then stained with E-cadherin and vimentin. Metastases showed no E-cadherin staining, despite strong E-cadherin staining in adjacent healthy lung tissue (Supplementary Figure S4B). Lung metastases were positive for vimentin, while the adjacent healthy lung tissue had very few vimentin-positive regions (Supplementary Figure S4B). Cultured cells from the lung metastases were also negative for E-cadherin and positive for vimentin and fibronectin (Supplementary Figure S4C). Cultured DT cells were used as a control for E-cadherin staining, and cultured AT3 cells were used as a negative control for E-cadherin staining and a positive control for vimentin and fibronectin (Supplementary Figure S4C). The results from the MET reporters and staining indicate that MET is a rare event during both AT3 tumorigenesis and formation of metastatic colonies in the lungs.

### Suicide reporters of MET

The MET reporters, along with staining of metastatic colonies, strongly suggested that MET was not required for metastatic invasion of the lungs. Yet, based on the experiments above, we cannot rule out that the rare MET events observed influenced the process and were required for colonization of metastases. To test whether or not the formation of macrometastases requires MET, we designed a ‘suicide reporter' in which MET triggers cell death via expression of an attenuated form of the A chain of diphtheria toxin (DipA). The reporter, E-cadDipIIIcI^2^, is the same as E-cadCreIIIcI^2^, except that the Cre ORF is replaced by a modified DipA ORF.^22^

**Figure 4:**
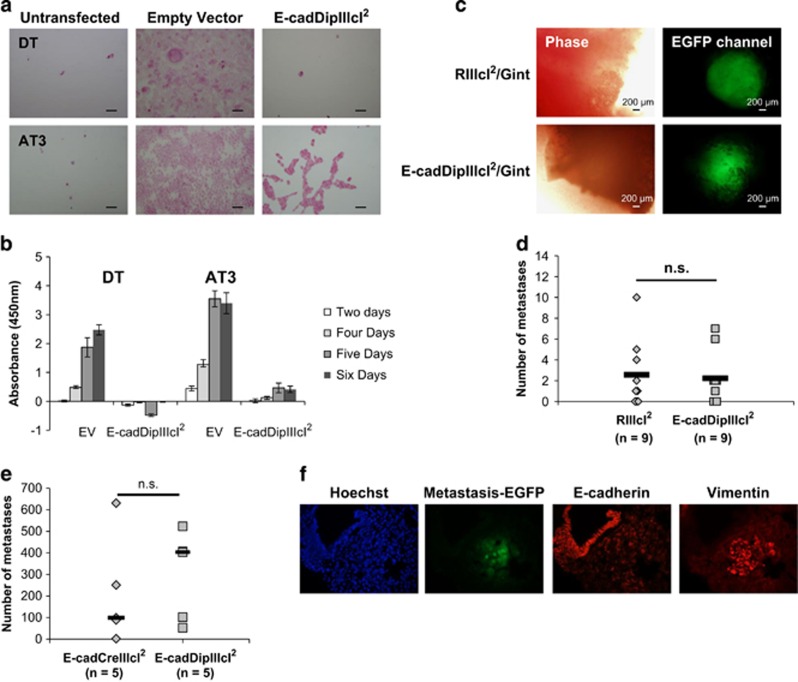
AT3 and DU145 cells metastasize via MET-independent pathways. (**a**) DTs and AT3 cells were left untransfected or transfected with an empty vector (pcDNA6) or E-cadDipIIIcI^2^ and subsequently selected with blasticidin. All untransfected DT and AT3 cells are killed by blasticidin, while DTs and AT3 cells transfected with an empty vector containing a blasticidin resistance marker survive blasticidin selection. Importantly, only mesenchymal AT3 cells transfected with the E-cadDipIIIcI^2^ suicide reporter grow out during blasticidin selection, while all epithelial DT cells with the reporter die. (**b**) Quantification of DT and AT3 growth during blasticidin selection by WST-1 cell growth assay. (**c**) Representative lung metastases from AT3 cells harboring RIIIcI^2^+Gint or E-cadDipIIIcI^2^+Gint. (**d**) No difference was observed in the number of macrometastases in each group. (**e**) Injection of DU145 E-cadCreIIIcI^2^ (as a control) or E-cadDipIIIcI^2^ cells led to formation of lung metastases with 100% penetrance. There was no difference in the number of metastases between control cells or cells with the suicide reporter. (**f**) DU145 E-cadDipIIIcI^2^ metastases lack E-cadherin expression and stain positive for vimentin.

To test the suicide reporters, DT and AT3 cells were transfected with either an empty vector control (pcDNA6) containing a blasticidin resistance gene or pcDNA6+E-cadDipIIIcI^2^. Two days after transfection, cells were treated with blasticidin. After 6 days of blasticidin selection, all mock-transfected DT and AT3 cells were killed, while both cell types transfected with an empty vector that conferred blasticidin resistance grew out efficiently (Figure 4a). There was, however, a remarkable difference between DT and AT3 cells transfected with E-cadDipIIIcI^2^. Although there were very few, if any, DT colonies, many AT3 colonies were detected (Figure 4a). Notably, AT3 cells containing the suicide reporter grew out at a slower rate than AT3 cells harboring an empty vector, likely because of the toxicity of even minute levels of DipA expression.

Selection for stable integrants was quantified using the WST-1 cell proliferation reagent and normalized to the absorbance from mock-transfected cells. Both DTs and AT3 cells transfected with the empty vector grew out quickly during selection, albeit at different rates (Figure 4b). DT cells transfected with the suicide reporter were negative when normalized to untransfected wells, which may be due to the combined toxicity of blasticidin and DipA expression in these cells (Figure 4b). AT3 cells transfected with the suicide reporter, on the other hand, produced colonies within 4 days of blasticidin selection (Figure 4b). In addition, we observed no difference in the size of primary tumors derived from cells harboring E-cadDipIIIcI^2^ compared with tumors from cells expressing RIIIcI^2^ (not shown).

AT3 cells containing Gint were stably transfected with either RIIIcI^2^ or E-cadDipIIIcI^2^, and 1 × 10^6^ cells injected into nude rats via the t.v. After 12 days, lungs were harvested and tested for the presence of the E-cadDipIIIcI^2^ reporter in the metastases using PCR. We were able to confirm the presence of the E-cadDipIIIcI^2^ reporter within 3/4 of AT3 lung metastases (Supplementary Figure S5A). Expression of DipA mRNA containing the IIIc exon in metastatic lung nodules was determined by RT–PCR using a forward primer within the 5' DipA exon and a reverse primer within the IIIc exon. DipIIIc mRNA was detected in 3/4 metastatic nodules, albeit at low levels when compared with AT3 E-cadDipIIIcI^2^ cells in culture (Supplementary Figure S5B). These results indicate that the suicide reporter remained within the cells during metastatic colonization.

To determine whether selective killing of cells undergoing MET reduced metastatic colonization in the lungs, the numbers of macrometastases from cells with RIIIcI^2^/Gint or E-cadDipIIIcI^2^/Gint were counted by epifluorescence microscopy. Figure 4c shows representative images of metastases from RIIIcI^2^/Gint and E-cadDipIIIcI^2^/Gint cells. There was no qualitative difference in the size or gross morphology of the metastases, with metastases from cells containing either the control or suicide reporter forming round, multicellular, secondary lung tumors (Figure 4c). Importantly, AT3 cells harboring RIIIcI^2^/Gint or E-cadDipIIIcI^2^/Gint formed equivalent numbers of metastases (Figure 4d).

To assess whether MET-independent metastatic colonization could be observed in human cancer cells, we also performed t.v. injections of DU145 human prostate adenocarcinoma cells harboring E-cadDipIIIcI^2^/Gint. We expected that this commonly used model of human adenocarcinoma may undergo MET during metastasis. The DU145 line features a predominantly mesenchymal phenotype, but has been shown to contain a small sub-population of epithelial-like cells.^23^ We first verified the expression of DipIIIc *in vitro* by sorting single cells and screening clones by RT–PCR. A total of 90% of DU145 clones expressed DipIIIc (Supplementary Figure S5C). Based on the high percentage of DipIIIc expression among clones, we opted to use the entire population of DU145s rather than individual clones. Consistent with the AT3 model, DU145 E-cadDipIIIcI^2^ cells did not undergo MET when metastasizing to the lungs (Figure 4e). DU145 cells harboring control reporters developed a median of 99 metastases, while DU145 E-cadDipIIIcI^2^/Gint cells developed a median of 403 metastases (Figure 4e). The DU145 E-cadDipIIIcI^2^ lung metastases lacked E-cadherin and expressed vimentin (Figure 4f). Overall, the results from lineage-tracing reporters and suicide reporters indicate that MET is not required for metastatic colonization of the lungs in the post-EMT AT3 and DU145 prostate cancer models.

### Suicide reporters reveal distinct metastatic pathways

**Figure 5:**
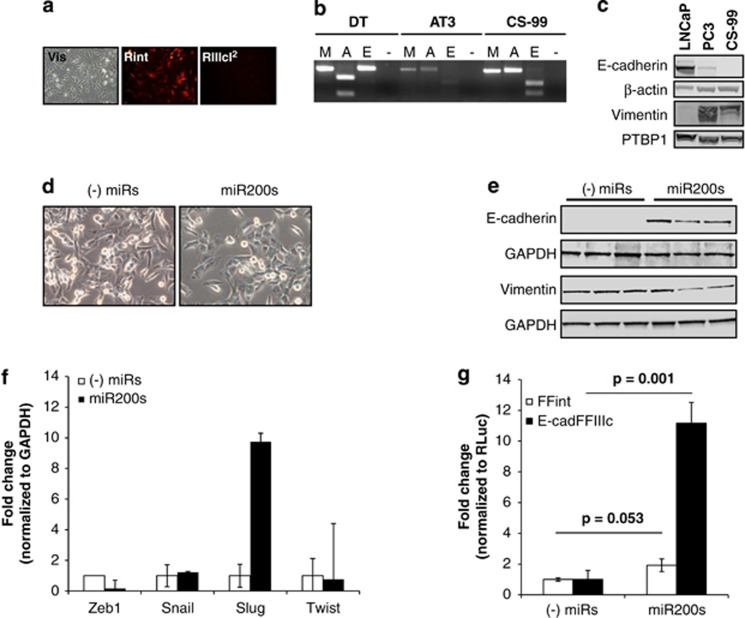
CS-99 human carcinosarcoma cells undergo MET *in vitro*. (**a**) Cultured CS-99 cells possess cell–cell contacts, interspersed with spindle-shaped cells. CS-99 cells transfected with Rint express DsRed, while cells transfected with RIIIcI^2^ express only background levels of DsRed. (**b**) CS-99 cells express FGFR2-IIIc mRNA. Epithelial DT and mesenchymal AT3 cells are included as controls for the IIIb and IIIc isoforms, respectively. For each cell type, RT–PCR products are mock digested (M), digested with the FGFR2 IIIb-specific enzyme, AvaI (A), or digested with the FGFR2-IIIc-specific enzyme, EcoRV (E). (**c**) CS-99 cells lack E-cadherin and express vimentin. Epithelial LNCaP and mesenchymal PC3 cells are included as controls. (**d**) Reverse transfection of miR200a, miR200b and miR200c into CS-99 cells leads to a morphological change consistent with MET; cells change shape from spindle-like, single cells to clusters of rounded cells. (**e**) Expression of miR200s in CS-99s induces upregulation of E-cadherin and loss of vimentin. (**f**). The miR200 targets, ZEB1 and Slug, are downregulated and upregulated, respectively. (**g**) Induction of MET in CS-99 cells has no effect on luciferase expression from a control reporter, FFint, but a significant increase in luciferase expression is observed from the E-cadFFIIIcI^2^ MET reporter.

We noted a number of similarities between the AT3 tumors and prostate carcinosarcomas, including their undifferentiated histology, mesenchymal-like gene expression profiles,^17^ aggressive nature and preference for metastasizing to the lungs and lymph nodes.^24^ Indeed, AT3 cells resemble the more poorly differentiated component of human carcinosarcomas in their histopathology (Supplementary Figure S6A), their likely epithelial origin and weak to negative co-expression of epithelial biomarkers, such as cytokeratin, and expression of vimentin (Supplementary Figure S6B). To understand whether MET-independent metastasis is a property of human carcinosarcomas, we tested our suicide reporters in the human uterine carcinosarcoma cell line, CS-99.^25^ Morphologically, CS-99 cells exhibit a spindle-like morphology, but will form cell–cell attachments at higher confluence (Figure 5a). CS-99s stably transfected with Rint, a plasmid encoding DsRed interrupted by a constitutively spliced intron, express DsRed; however, CS-99s transfected with RIIIcI^2^ exhibit only low levels of DsRed expression (Figure 5a), which is consistent with FGFR2 exon IIIc inclusion and a mesenchymal phenotype. We also characterized the expression of endogenous FGFR2 isoforms in CS-99s by RT–PCR (Figure 5b). Similar to the results from the RIIIcI^2^ reporter, CS-99 cells exclusively express FGFR2-IIIc mRNA (Figure 5b). CS-99s lack E-cadherin and express vimentin (Figure 5c). Together, these data verify the mesenchymal-like phenotype and biomarker expression of the CS-99 cell line.

Although CS-99s are mesenchymal-like, we noted that these cells can readily undergo MET *in vitro*. Combined ectopic expression of microRNA (miR)200a, miR200b and miR200c in CS-99s led to a morphological change consistent with MET (Figure 5d). Expression of miR200s also led to dramatic upregulation of E-cadherin and a reproducible but modest reduction in vimentin at the protein level (Figure 5e). In addition, qPCR revealed downregulation of the miR200 target and EMT transcription factor, Zeb1, but no effect on Twist1 and Snail in cells transfected with miR200s, and a surprising increase in the levels of Slug mRNA (Figure 5f). The morphological change and biomarker profile suggests that CS-99s undergo a partial MET event *in vitro*. We used this MET induction system to test whether our reporters could detect these MET-like events in CS-99s. To do this, we induced MET with miR200s and subsequently transfected cells with a control reporter (FFint) or the E-cadFFIIIcI^2^ reporter and quantified the levels of luciferase production. Although MET induction had little effect on the FFint reporter, CS-99s transfected with E-cadFFIIIcI^2^ exhibited significantly higher luminescence relative to control cells when an MET was partially induced by expression of miR200s (Figure 5g). These results provide proof-of-principle that our reporter strategy is not only capable of detecting steady-state phenotypes, but can also identify toggling of cells between mesenchymal-like and epithelial-like states.

To ask whether CS-99 carcinosarcoma cells require MET during metastasis, we stably transfected the E-cadDipIIIcI^2^ suicide reporter followed by Gint, which marked these cells with EGFP. We selected by RT–PCR analysis three clonal cell lines (clone 23, clone 27 and clone 33) that contained E-cadDipIIIcI^2^ and expressed DipIIIc (Supplementary Figure S5C). Because the CS-99 cell line had never been used in any *in vivo* study, and as such, we had no indication of the metastatic penetrance *in vivo* we designed all of our experiments to include CS-99 cells that lacked the E-cadDipIIIcI^2^ reporter. To this end, we stably transfected CS-99s with Rint and sorted DsRed-expressing cells by fluorescence activated cell sorting. Next, to control for the presence of Gint in E-cadDipIIIcI^2^/Gint cells, we also transfected Gint and sorted DsRed+/Gint− cells by fluorescence activated cell sorting.

**Figure 6:**
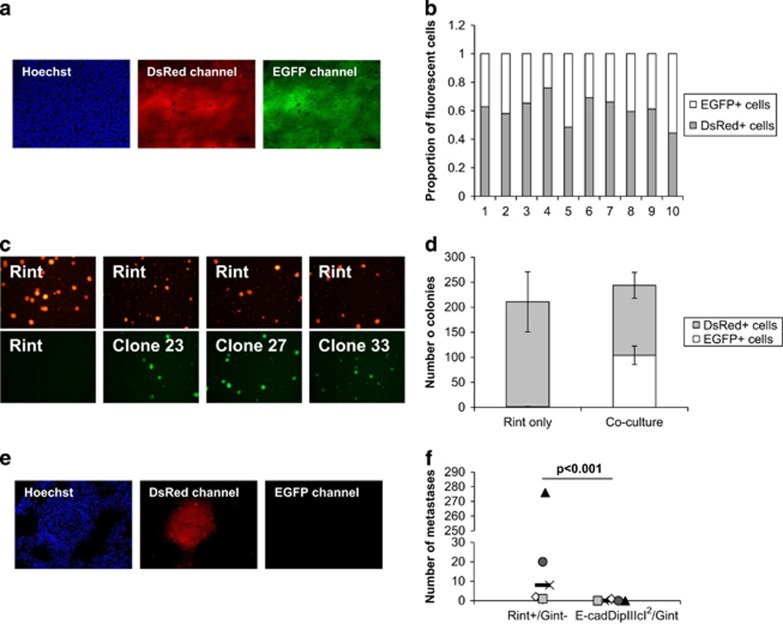
CS-99 metastasize via MET-dependent pathways. (**a**) Rint+/Gint− CS-99 cells were mixed 1:1 with CS-99 clones harboring E-cadDipIIIcI^2^/Gint and injected s.c. CS-99 Rint+/Gint− and E-cadDipIIIcI^2^/Gint+ cells formed primary tumors with 100% penetrance. (**b**) Flow-cytometric analysis of the proportion of DsRed+ and GFP+ cells within primary tumors indicates that all primary tumors contain both DsRed+ control cells and E-cadDipIIIcI^2^/Gint+ cells. (**c**) Representative images and (**d**) quantification of CS-99 Rint+/Gint− control cells and E-cadDipIIIcI^2^/Gint+ suicide reporter cells grown in soft agar. (**e**) Rint+/Gint− CS-99 cells were mixed 1:1 with CS-99 clones harboring E-cadDipIIIcI^2^/Gint and injected via the t.v. With the exception of a single GFP+ colony, all metastases were DsRed+ and lacked EGFP expression. (**f**) Quantification of CS-99 EGFP+ and DsRed+ lung metastases. Each unique symbol represents the number of metastases from a single animal. The median number of Rint+/Gint− and E-cadDipIIIcI^2^/Gint+ metastases is indicated by a solid black bar.

Using these clonal lines, we asked whether harboring the E-cadDipIIIc^2^ would affect CS-99 growth rates. *In vitro* WST-1 cell proliferation assays indicated that the E-cadDipIIIcI^2^/Gint clones and Rint+/Gint− cells grew at equal rates (Supplementary Figure S8A). To test whether or not MET was required for growth *in vivo*, we co-injected a 1:1 ratio of Rint+/Gint− control cells and E-cadDipIIIcI^2^/Gint+ cells s.c. into the flanks of nude mice. Palpable tumors formed within 5–7 days. Analysis of the proportions of control cells and cells with the suicide reporter revealed that 100% of primary tumors contained substantial proportions of both cell types (Figure 6a). We observed only a very slight decrease in the proportion of E-cadDipIIIcI^2^/Gint cells relative to control Rint+/Gint− cells (Figure 6b). Indeed, cells harboring the suicide reporter cells formed colonies in soft agar only slightly less efficiently than control cells (Figures 6c and d). We concluded from this that MET was not required for growth and primary tumor formation *in vitro* or *in vivo*.

To test the requirement for MET in late stages of the metastatic cascade in parallel with experiments above with AT3 and DU145 cells, we co-injected DsRed+/Gint− cells with E-cadDipIIIcI^2^/Gint+ clones via the t.v. of Balb/c nu mice (*n*=3 for clone 23, *n*=3 for clone 27 and *n*=4 for clone 33). This strategy, which includes control and experimental cells in the same injection, controls for technical issues related to the t.v. injections. Using this design, we were able to interrogate the importance of MET in metastasis, focusing on those animals for which the t.v. injections themselves worked properly. After four weeks, 5/10 animals that were co-injected with control cells and E-cadDipIIIcI^2^/Gint+ cells had lung metastases. We evaluated the morphology and biomarker expression in the lung metastases and for comparison also in the primary tumors described above. Both primary tumors and metastases presented as undifferentiated sheets of cells with little to no E-cadherin expression and high levels of vimentin and Zeb1 (Supplementary Figure S8B–D). Of the animals with metastases, all animals had Rint+ metastases. The number of Rint+ metastases ranged from 1 to 276; with a median of 8 metastases per animal (Figures 6e and f). In contrast, there was only one Gint+ metastasis observed (Figure 6f). All other animals completely lacked E-cadDipIIIcI^2^/Gint+ cells, despite the presence of between 1 and 276 metastases from control Rint+/Gint− cells (Figure 6a). In stark contrast to our observations for growth in primary tumors, we conclude that CS-99 requires MET to take hold and/or colonize the metastatic niche.

## Discussion

During carcinoma progression, EMT is generally accepted as a means by which cancer cells gain invasive capabilities and break free from the confines of the primary tumor.^2, 26^ Several investigations have suggested that MET may be important for metastatic colonization by reactivating cell signaling pathways and/or facilitating attachment to heterologous cells within the healthy tissue.^10, 11, 27^ Like these previous studies, the present work also suggests that CS-99 human carcinosarcoma cells require MET to efficiently metastasize in the lungs. Yet, our data also indicate that other, post-EMT cancers can metastasize through a mechanism that appears to be completely independent of MET.

The finding that MET appears to be critically important for metastasis of some cancers, while others have no apparent need for this transition was predicted by Brabletz,^3^ who speculated that some cancers will be plasticity dependent while other cancers will metastasize by way of one or more plasticity-independent pathways. Importantly, our data provide experimental evidence for both the MET-dependent and MET-independent routes to metastasis.

In summary, our combinatorial reporters identified, for the first time, plasticity-dependent and -independent mechanisms of metastasis. Interestingly, the present study suggests that the same cancer type, and maybe even cells within a single human tumor, may be capable of metastasizing via different routes. Paraphrasing Weinberg,^28^ each cancer cell is an experiment of nature sampling solutions and usually finding several that can lead to clinically significant metastases.

## Materials and methods

### Analysis of MET biomarkers in patient bone metastases

Gene expression of EMT markers was compared between primary prostate cancer samples and bone metastases^19^ (GSE32269) using GEO2R.

### Cloning and expression of reporters

Both the RIIIcI^2^ ^20^ and Gint^29^ reporters have been described previously. To construct the lineage-tracing reporters, the DsRed ORF from RIIIcI^2^ was replaced with the Cre ORF from Addgene plasmid 11916,^30^ separated into two pseudo-exons, with the first Cre pseudo-exon containing amino acids 1–161 (bp 1–483) of the ORF and the second Cre pseudo-exon containing amino acids 162–343 (bp 484–1032) of the ORF. Cre pseudo-exon one was inserted via *Hin*dIII and *Xba*I, and pseudo-exon two was inserted using *Apa*I and *Age*I. The CMV promoter of RIIIcI^2^ was replaced with bp 638–1028 of the human E-cadherin promoter to create E-cadCreIIIcI^2^. The RG plasmid (a gift of Dr Rita DeGasperi) was subcloned into pcDNA3.1+ (G418) downstream of the CMV promoter via 5' *Nhe*I and 3' *Apa*I. The E-cadDipIIIcI^2^ reporter was created in a similar manner to that of E-cadCreIIIcI^2^. The DipA pseudo-exon one consists of amino acids 1–24 (bp 1–72), and pseudo-exon two comprises amino acids 25–193 (bp 73–588). The FFint and FFIIIcI^2^ reporters have been described previously.^16^ E-cadFFint and E-cadFFIIIcI^2^ were constructed by replacing the CMV promoter from FFint and FFIIIcI^2^ with the E-cadherin promoter described above (a kind gift of Drs Jennifer Yori and Ruth Keri^31^).

### Tumor cell injections and processing of primary and metastatic tumors

AT3 cells (5 × 10^5^ cells/200 μl) were injected s.c. into the left flanks or via t.v. (1 × 10^6^ AT3 cells/200 μl) into Copenhagen or nude rats (Charles River, Wilmington, MA, USA). CS-99 cells (2 × 10^6^/200 μl) were injected s.c. into the flanks of female Balb/c nu mice. CS-99 (1 × 10^6^/200 μl) and DU145 (5 × 10^6^ cells/200 μl) cells were injected via t.v. into Balb/c nu mice. For cryosectioning and staining, tissues were flash frozen in OCT compound and stored at −80 °C until use. For RNA and DNA extraction, lung metastases were stored in RNA Later at −80 °C or frozen at −20 °C, respectively, until use.

### Immunofluorescence staining, imaging and flow cytometry

Tumor and lung cryosections were prepared using a Microm HM 550 cryotome (Thermo Scientific, Waltham, MA, USA) and affixed to glass slides for staining. Fluorescence images were captured using an Olympus IX 71 epifluorescence microscope (Olympus America, Center Valley, PA, USA) with a DP70 digital camera and processed with the CellSens software (Olympus America). Antibody dilutions and catalog numbers are provided in Supplementary Table S9. All flow-cytometry analyses and sorts were performed by the Duke University Flow Cytometry Shared Resource. The percentage of MET-like events in cultured cells, tumors and metastases was calculated by dividing the number of EGFP+ cells from the total number of fluorescent cells in each specimen.

### Nucleic acid extraction and PCR

DNA and RNA extraction, PCR and RT–PCR were carried out following standard protocols. Primer sequences are listed in Supplementary Table S10.

### MET induction in CS-99 cells

Negative control miRs (Life Technologies, Thermo Scientific; 4464058) or a combination of miRNA 200a (Life Technologies, Thermo Scientific; MC10991), miR200b (Life Technologies, Thermo Scientific; MC10492) and miR200c (Life Technologies, Thermo Scientific; MC11714) *mir*Vana miRNA mimics were reverse transfected into CS-99 cells using RNAiMAX. Negative control miRs (150 nm final concentration) or miR200 mimics (50 nm final concentration of each miR200) in 50 μl Opti-MEM were mixed with 1 μl of RNAiMAX in 50 μl of Opti-MEM and incubated at room temperature for 20 min. RNA/lipid complexes were plated in 24-well format, and 25 000 cells were added in 400 μl of DMEM+10% FBS+1% penicillin/ streptomycin. Transfections were incubated overnight at 37 °C, and media was changed the next day. After 48 h, 400 ng of FFint or E-cadFFIIIc was transfected, along with 400 ng of a Renilla luciferase control reporter. Two days later, cells were lysed in 1 × passive lysis buffer, and luciferase output was read in a SpectraMax M3 plate reader (Molecular Devices, Sunnyvale, CA, USA).

### Statistical analyses

Differences between luciferase outputs from the various reporters (Supplementary Figure S2) were analyzed using one-way analysis of variance with Tukey's *post hoc* correction. qRT-PCR results were tested for significance using a Student's *t*-test. MET reporter luciferase assays in CS-99 cells were analyzed using a Student's *t*-test. Percentages of MET from the lineage-tracing experiments and the number of metastases from suicide reporter experiments were analyzed by the Kruskal–Wallis test. Differences in growth rates between CS-99 E-cadDipIIIcI^2^ clones and Rint+/Gint− cells and differences in the number of colonies formed in soft agar were analyzed by analysis of variance. All analyses were performed using JMP (version 9.0; SAS, Cary, NC, USA).
