# Supplementary figures and images for: Distinct routes to metastasis: plasticity-dependent and plasticity-independent pathways
Source: Oncogene. 2016 Jan 11;35(33):4302–11. doi: 10.1038/onc.2015.497 (PMC4940344; doi:10.1038/onc.2015.497)

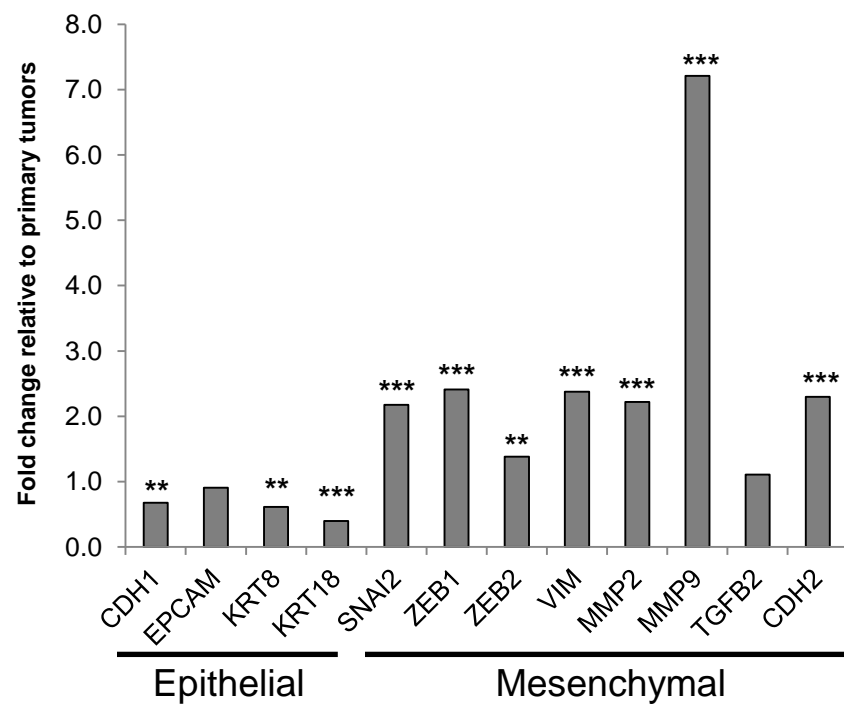

Supplement: Supplementary Figure 1 [file onc2015497x1.pdf]

**A**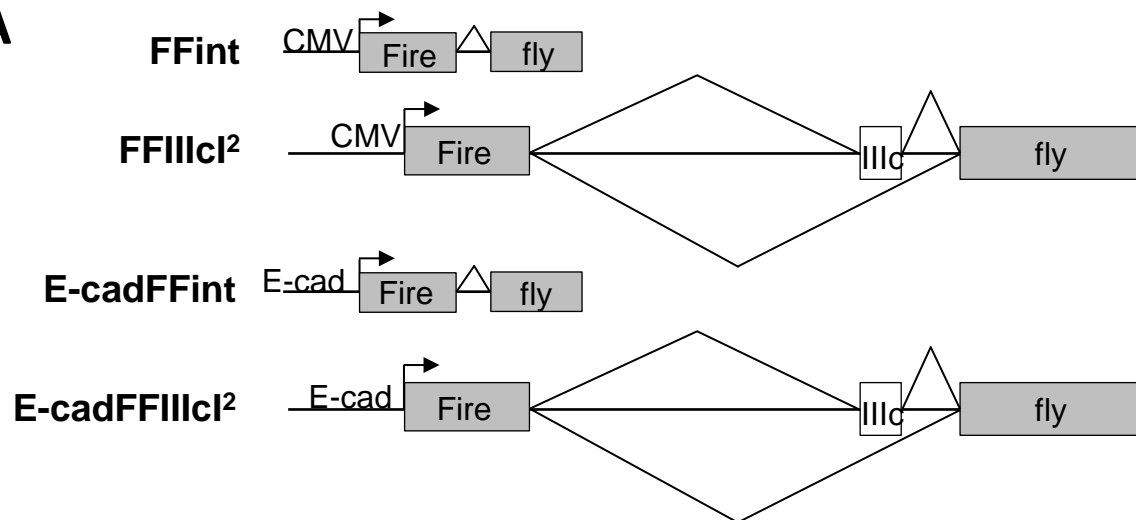**B**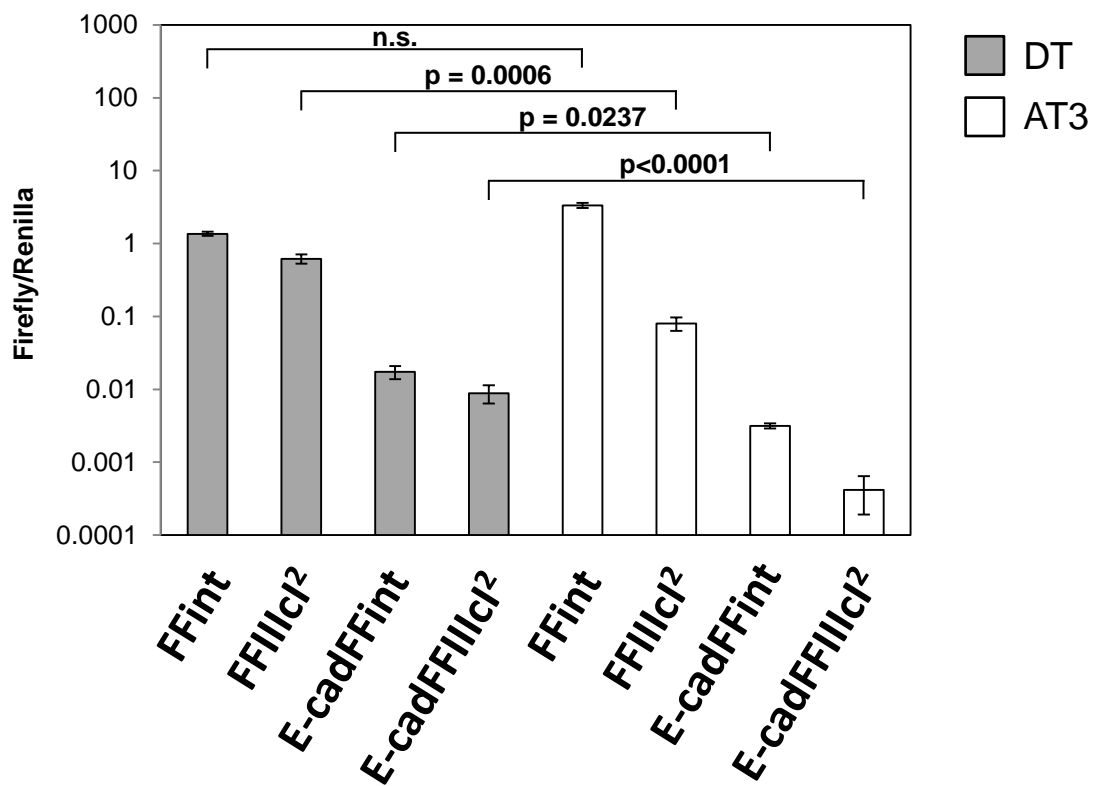

Supplement: Supplementary Figure 2 [file onc2015497x2.pdf]

A

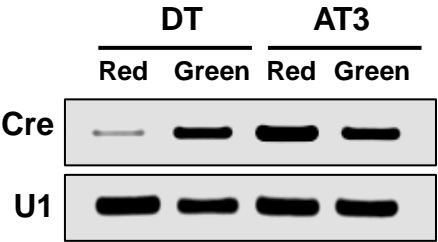

B

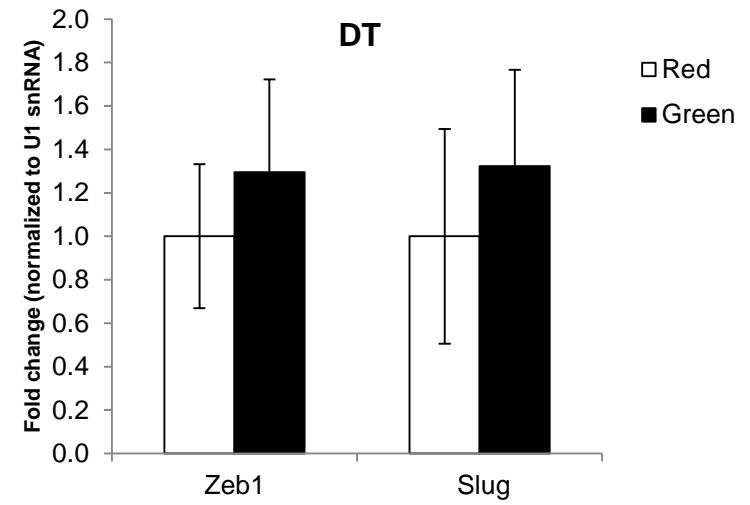

Supplement: Supplementary Figure 3 [file onc2015497x3.pdf]

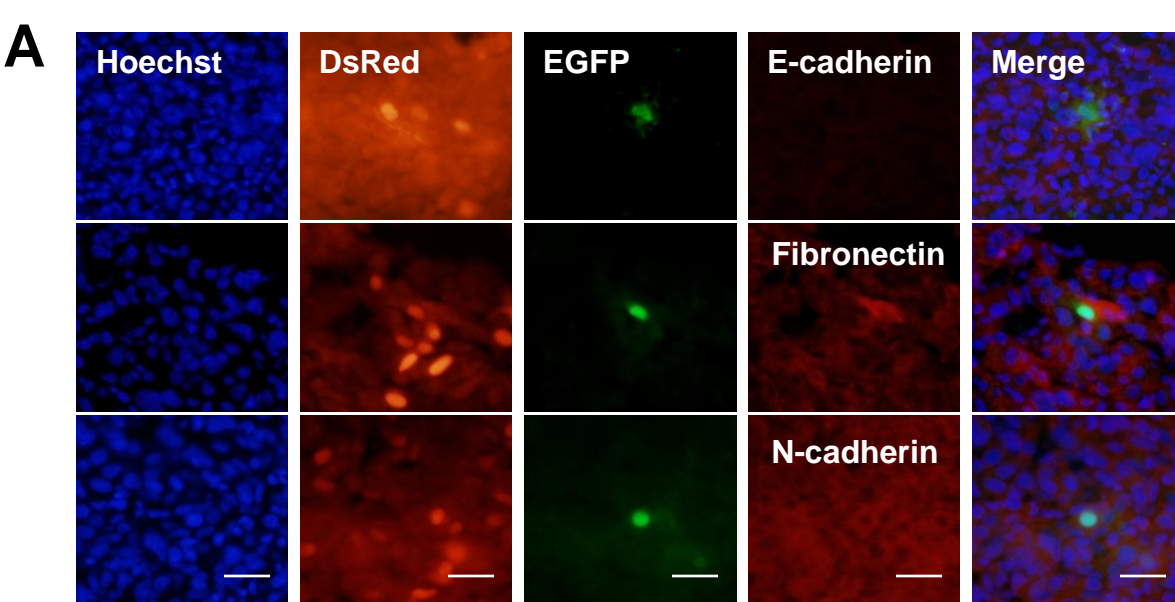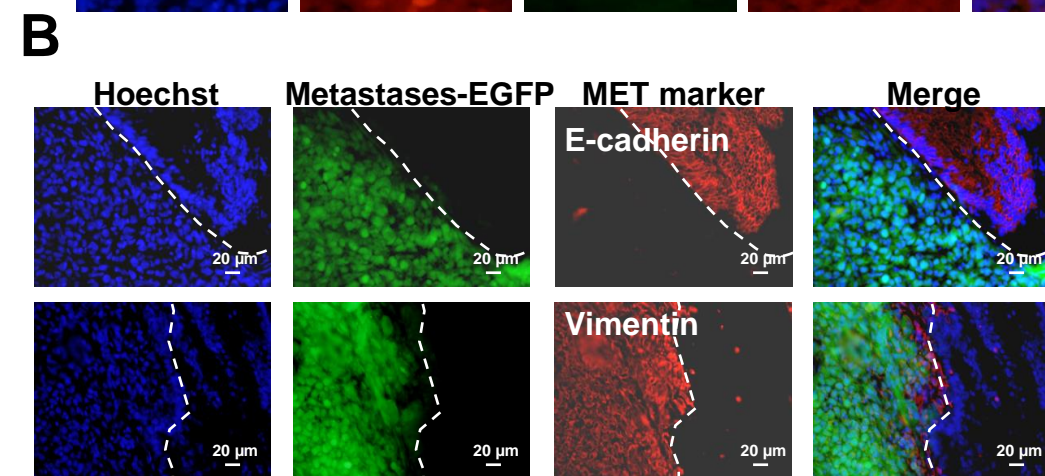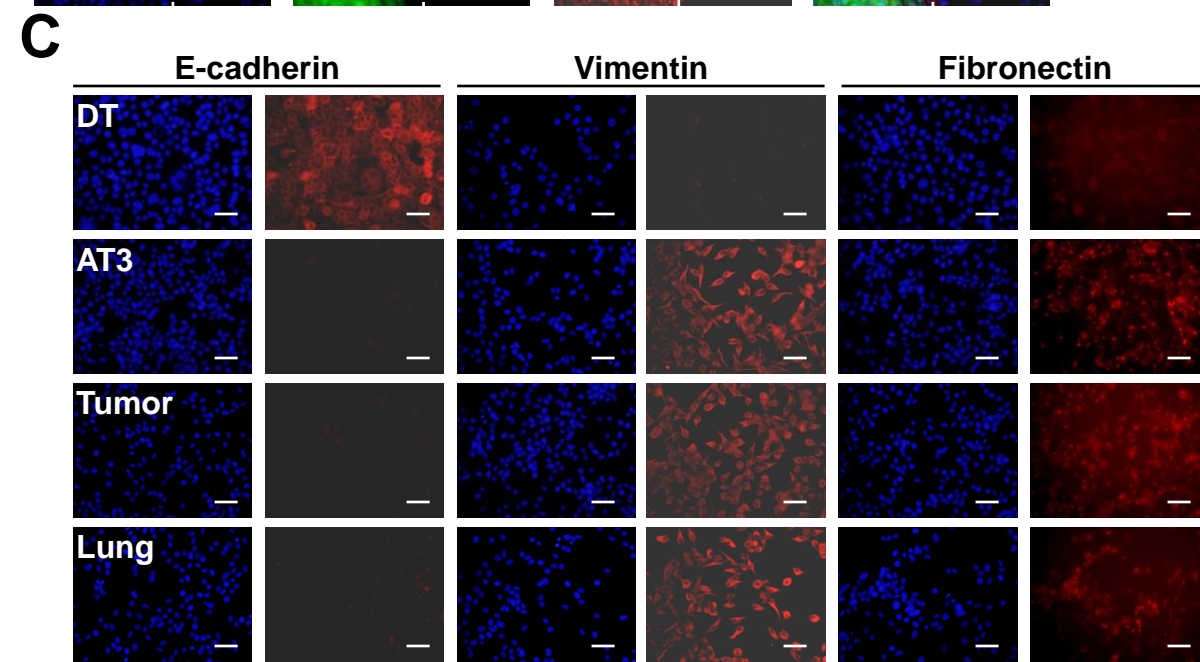

Supplement: Supplementary Figure 4 [file onc2015497x4.pdf]

A

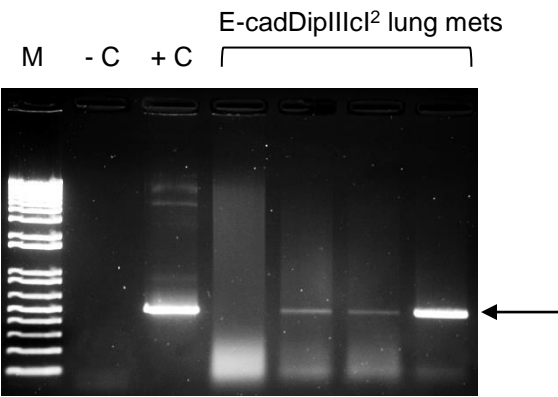

B

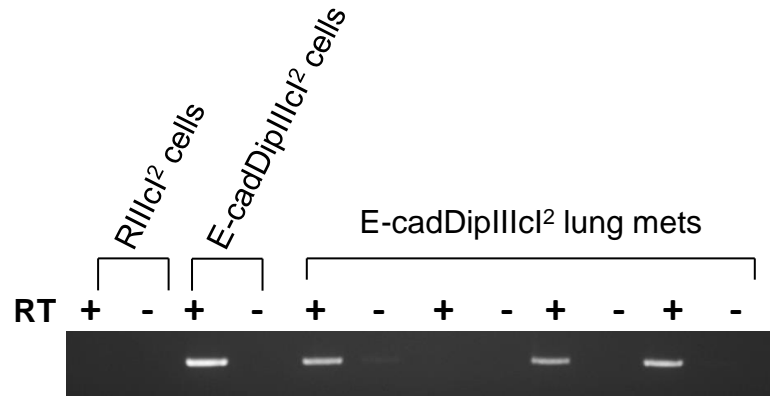

C

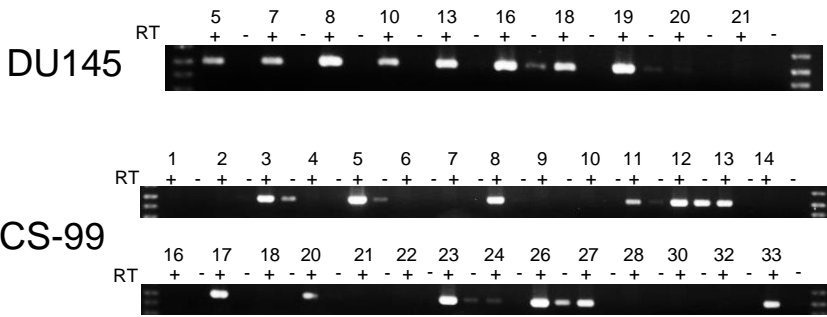

Supplement: Supplementary Figure 5 [file onc2015497x5.pdf]

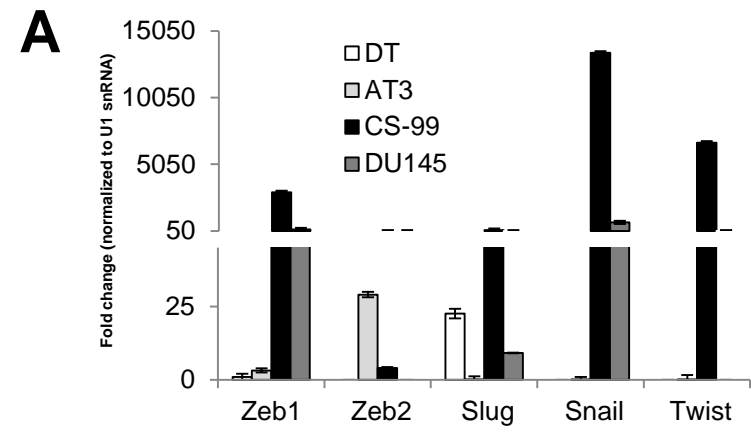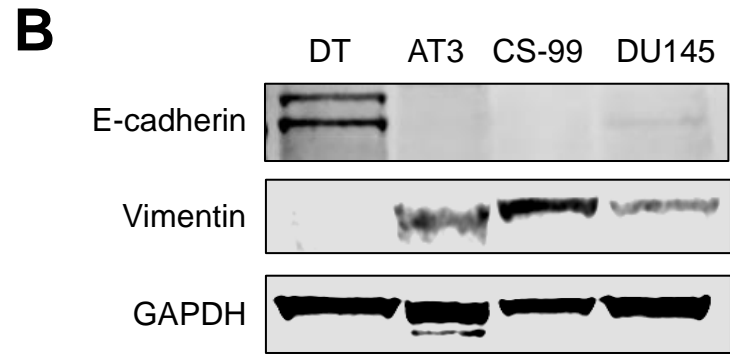

Supplement: Supplementary Figure 7 [file onc2015497x7.pdf]

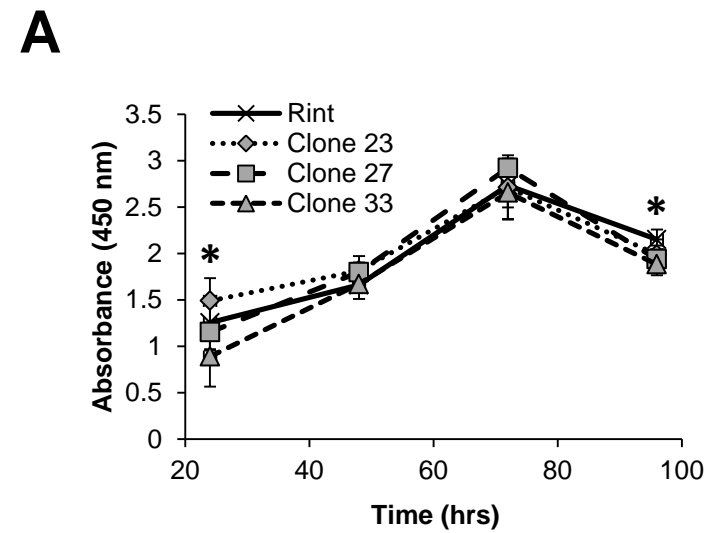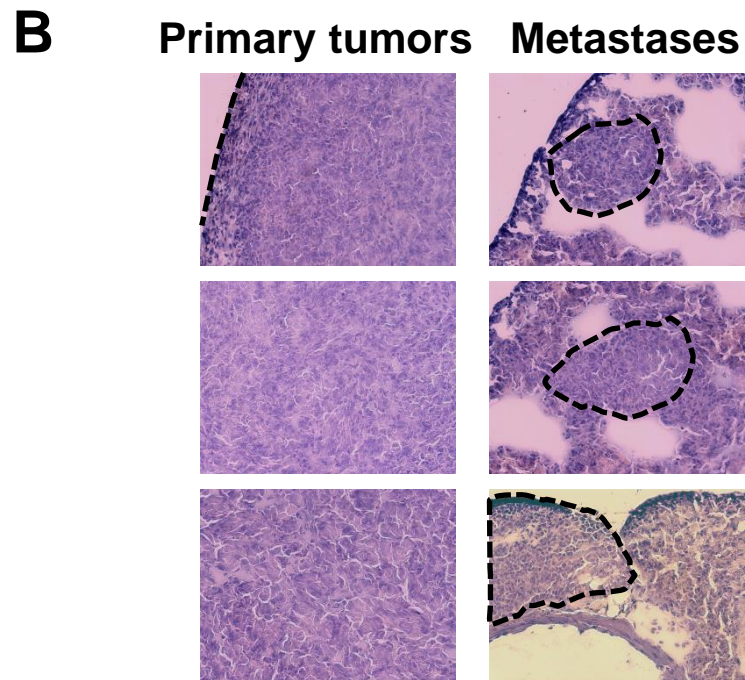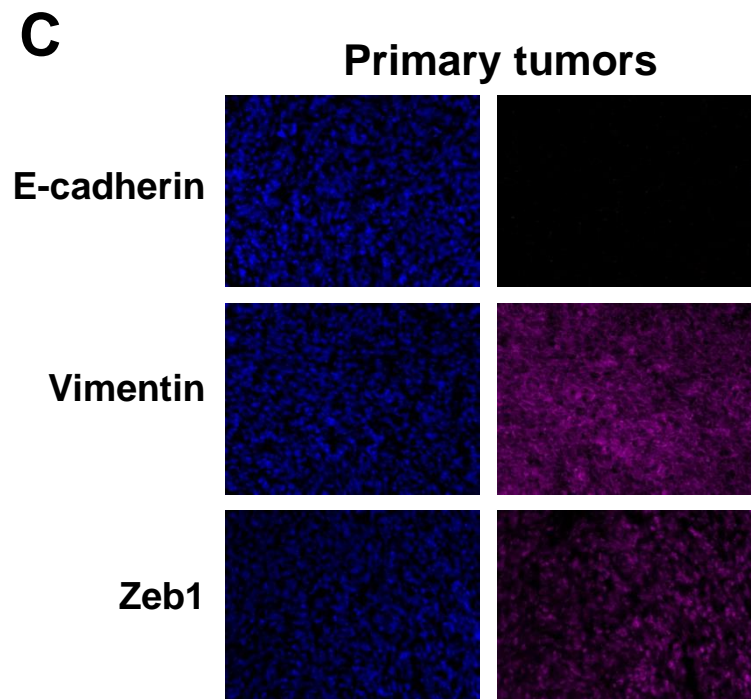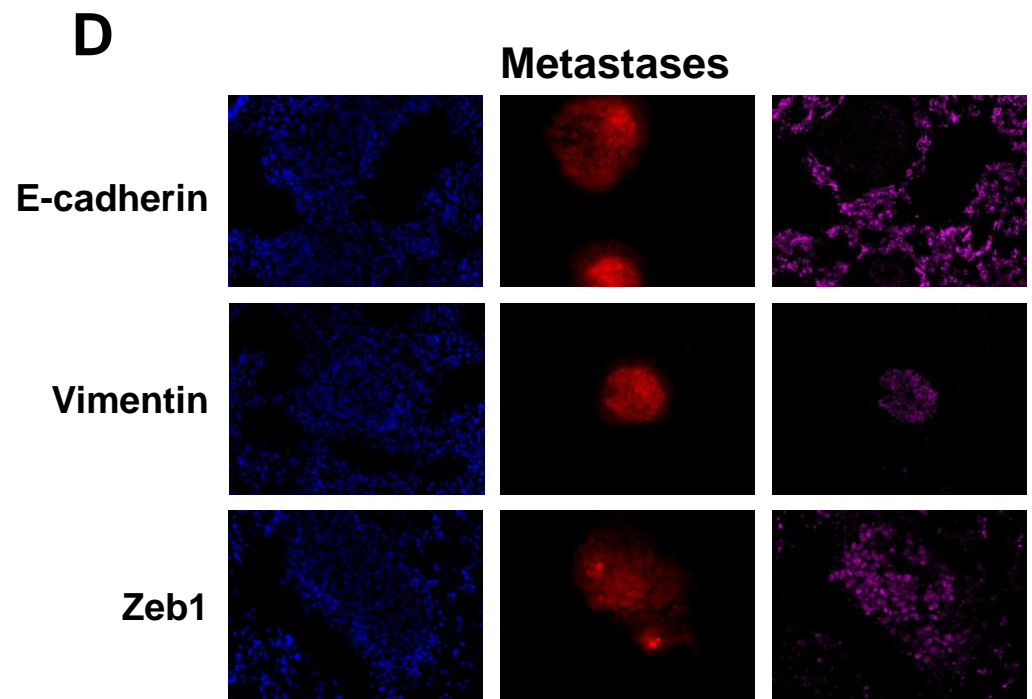

Supplement: Supplementary Figure 8 [file onc2015497x8.pdf]
