# Supplementary material for: Distinct routes to metastasis: plasticity-dependent and plasticity-independent pathways
Source: Oncogene. 2016 Jan 11;35(33):4302–11. doi: 10.1038/onc.2015.497 (PMC4940344; doi:10.1038/onc.2015.497)
Supplement: Supplementary Figure 6 [file onc2015497x6.pdf]

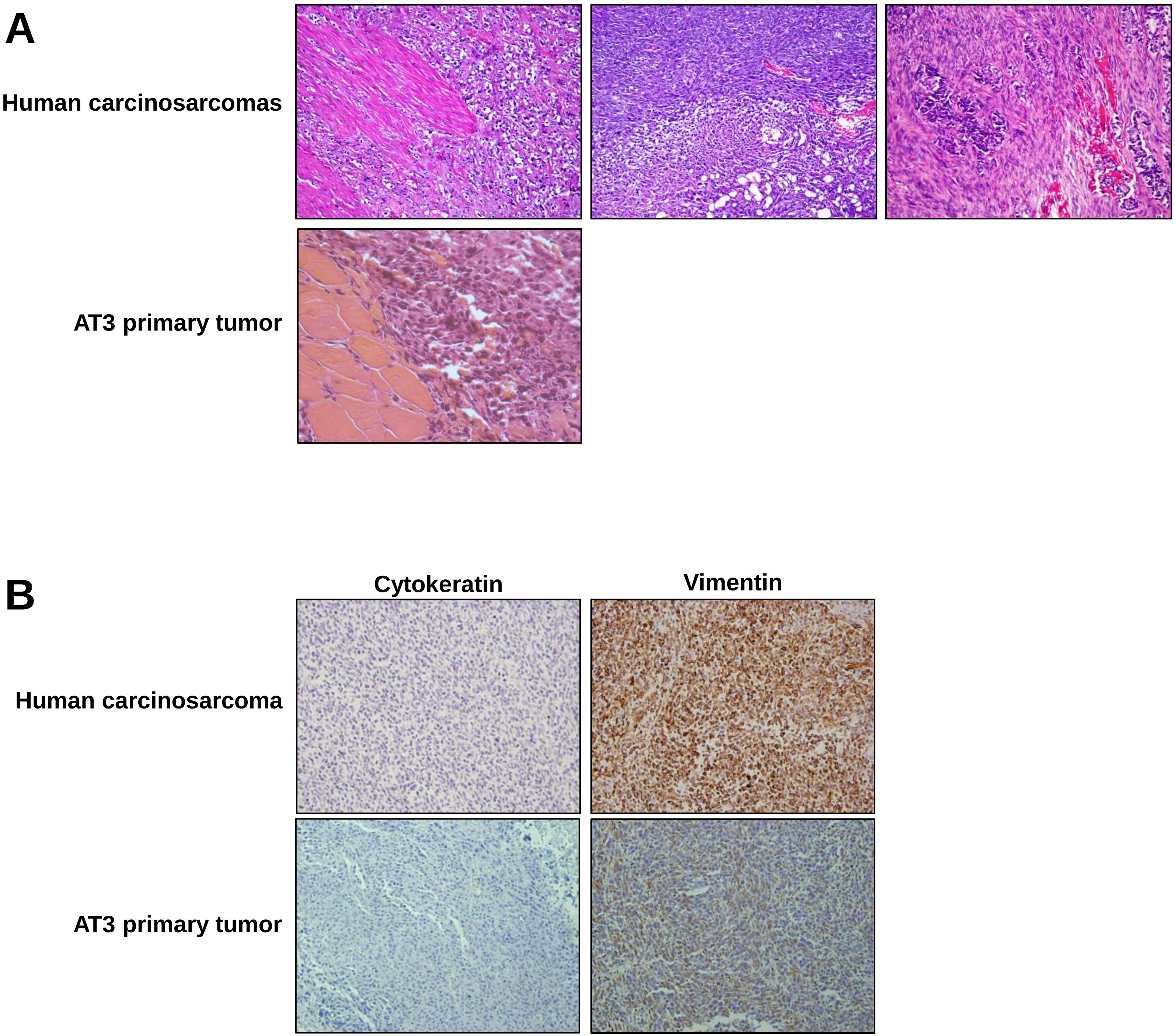
A
Human carcinosarcomas
AT3 primary tumor
B
Cytokeratin
Vimentin
Human carcinosarcoma
AT3 primary tumor
