# Supplementary material for: Distinct routes to metastasis: plasticity-dependent and plasticity-independent pathways
Source: Oncogene. 2016 Jan 11;35(33):4302–11. doi: 10.1038/onc.2015.497 (PMC4940344; doi:10.1038/onc.2015.497)
Supplement: Supplementary Table 1 [file onc2015497x9.docx]

| **Antibody** | **Company** | **Catalog Number** | **Dilution** |  |
| --- | --- | --- | --- | --- |
| mouse anti-E-cadherin | BD Biosciences | cat. #610181 | 1:1,000 | |
| rabbit anti-E-cadherin | Cell Signaling | cat. #3195S | 1:250 | |
| mouse anti-vimentin | ABD Serotec | cat. #MCA862 | 1:500 | |
| mouse anti-cellular fibronectin | Sigma | cat. #F6140 | 1:500 | |
| mouse anti-N-cadherin | BD Biosciences | cat. #610921 | 1:1,000 | |
| mouse anti-β-actin | Santa Cruz | cat. #sc-47778 | 1:4,000 | |
| rabbit anti-PTBP1 | Wagner et al. 1999 |  | 1:4,000 | |
| rabbit anti-GAPDH | Abcam | cat. #ab9485 | 1:2,000 | |
