# Supplementary material for: Distinct routes to metastasis: plasticity-dependent and plasticity-independent pathways
Source: Oncogene. 2016 Jan 11;35(33):4302–11. doi: 10.1038/onc.2015.497 (PMC4940344; doi:10.1038/onc.2015.497)
Supplement: Supplementary Table 2 [file onc2015497x10.docx]

| **Primer** | **Sequence (5’ to 3’)** | **Description/use** |
| --- | --- | --- |
| DipA FOR | CCT CAG ACT AAA CCT GGT TAT GTA GAT TCC ATT C | Used to amplify DipA within genomic DNA of lung metastases |
| DipA REV | GAT TTC CTG CAC AGG CTT GAG CCA TAT AC | Used to amplify DipA within genomic DNA of lung metastases |
| Cre FOR | GAA CCT GAT GGA CAT GTT CAG G | Used to amplify Cre |
| Cre REV | AGT GCG TTC GAA CGC TAG AGC CTG T | Used to amplify Cre |
| DipIIIc FOR | GGG CGC TGA TGA TGT TGT TGA TTC | Used to amplify exon IIIc-containing DipA transcripts |
| DipIIIc REV | TAC GCT TAA CGC TTT CGC CTG TTC | Used to amplify exon IIIc-containing DipA transcripts |
| hFGFR2 FOR | GCA AGG TTT ACA GTG ATG CCC AGC C | Used to amplify human FGFR2 in CS-99s |
| hFGFR2 REV | GGA TGA CTG TTA CCA CCA TAC AGG CGA T | Used to amplify human FGFR2 in CS-99s |
