# Supplementary material for: Distinct routes to metastasis: plasticity-dependent and plasticity-independent pathways
Source: Oncogene. 2016 Jan 11;35(33):4302–11. doi: 10.1038/onc.2015.497 (PMC4940344; doi:10.1038/onc.2015.497)
Supplement: Supplementary Information [file onc2015497x11.docx]

**Supporting Text**

**Supplementary Figure Legends**

**S1. Examining MET biomarkers in prostate cancer bone metastases. A.** Bone metastases from patients with prostate cancer have significantly lower levels of CDH1, KRT8, and KRT18, and significantly higher expression of numerous mesenchymal biomarkers, including Slug (SNAI2), Zeb1, Zeb2, vimentin (VIM), MMP2, MMP9, and CDH2. Genes that were unavailable in the data set are indicated by N/A. *: p<0.05, **: p<0.01, ***: p< 0.001. **~~B.~~** ~~Heterogeneity of MET biomarkers E-cadherin (CDH1) and vimentin (VIM) in human bone metastases. Normalized gene expression values are plotted for each metastatic sample (linear scale). The median value of CDH1 in primary tumors is indicated by the dashed line, and the median value of VIM in primary tumors is indicated by the solid line.~~

**S2. Combinatorial reporter control enhances discrimination between cell types. A.** To test whether combinatorial control of reporters has an additive effect on segregating different cell types, four constructs were created. The FFint and FFIIIcI^2^ reporters are identical to Gint and RIIIcI^2^, respectively, except that fluorescent proteins are replaced by the firefly luciferase ORF. The E-cadFFint and E-cadFFIIIcI^2^ reporters are identical to FFint and FFIIIcI^2^, respectively, except that the CMV promoter is substituted for the E-cadherin promoter. **B.** All four reporters were transfected into epithelial DT and mesenchymal AT3 cells along with a constitutively active *Renilla* luciferase plasmid. Splicing alone and promoter control alone provide seven-fold and four-fold discrimination, respectively, between cell types; however, the combined use of splicing and promoter control increases the difference in luciferase expression to 53-fold. Bars not connected by the same number are significantly different.

**S3. Validation of MET lineage tracing reporters. A.** PCR for Cre in DT and AT3 E-cadCreIIIcI^2^ cells sorted for EGFP or DsRed shows low expression of Cre in EGFP- DT cells and enrichment of Cre in all other cells. **B.** There is no difference in the expression of EMT transcription factors Zeb1 and Slug in DT cells harboring E-cadCreIIIcI^2^ and sorted for EGFP or DsRed.

**S4. AT3 metastases maintain a mesenchymal biomarker profile. A.** Immunohistochemical staining of AT3+RG+E-cadCreIIIcI^2^ tumor sections suggests that MET is transient, with EGFP expressing cells returning to a mesenchymal phenotype. Sections were stained with the epithelial marker E-cadherin, the mesenchymal markers fibronectin and N-cadherin. **B.** To verify that MET is rare in lung metastases, AT3 lung metastases were labeled with EGFP and stained for E-cadherin and vimentin. Lung metastases do not express the epithelial marker E-cadherin despite robust expression of E-cadherin in adjacent healthy lung. Conversely, AT3 lung metastases express high levels of the mesenchymal marker vimentin (red). **C.** Cultured AT3 cells from lung explants have low E-cadherin expression and high vimentin and fibronectin expression. Cultured epithelial DTs and AT3 cells were used as controls. Scale bars = 50 µm unless otherwise indicated.

**S5. Detection of the Diphtheria toxin gene and transcript in AT3 lung metastases. A.** A total of 75% (3/4) AT3 metastatic lung nodules were positive for the E-cadDipIIIcI^2^ reporter (black arrow). M: 1Kb+ molecular weight marker (Invitrogen); -C: no template control; +C positive control (E-cadDipIIIcI^2^ plasmid); lanes 4-7: DNA from E-cadDipIIIcI^2^ lung metastases. **B.** RT-PCR of exon IIIc-containing DipA transcripts in AT3 metastatic lung nodules. A total of 75% (3/4) of lung macrometastases expressed DipIIIc transcripts (black arrows). M: molecular weight marker (same as E above); -C: negative control (AT3 cultured cells containing RIIIcI^2^); +C: positive control (AT3 cultured cells containing E-cadDipIIIcI^2^); lanes 6-15: AT3 metastatic lung nodules. **C.** Analysis of DU145 and CS-99 E-cadDipIIIcI^2^ clones by RT-PCR. The clone number is listed above each lane. Molecular weight markers are included flanking the samples on each of the gels.

**S6. Histopathologic comparison of AT3 primary tumors and human carcinosarcomas.** **A.** Hematoxylin and eosin staining of human carcinosarcomas and AT3 primary tumors revealed that both tumors are poorly-differentiated while many areas show lack of epithelial differentiation on H&E stains. Images from each of three human carcinosarcoma samples are shown. Like the AT3 tumors, the human carcinosarcoma specimens are poorly-differentiated, with frequent foci of necrosis, high mitotic and apoptotic rates, and extensive invasion of adjacent structures. Yet, unlike the AT3 tumors and metastases, all three human tumors appeared to be biphasic, with part of the human tumors having a more spindled cytology and a fasciculated architecture suggestive of mesenchymal differentiation; other areas of these neoplasms were more poorly-differentiated, with features more like those of the AT3 cell line-derived tumors (vesiculated nuclei with nucleoli, and abundant cytoplasm). **B.** AT3 tumors were negative for pan-cytokeratin staining; two of the primary human tumors showed weak, focal cytokeratin staining in the more poorly-differentiated areas and the third was negative for cytokeratin. The human primary carcinosarcomas also displayed patchy vimentin staining throughout, as did the AT3 primary tumors. These data suggest that the AT3 tumors most closely resemble the poorly-differentiated component of carcinosarcomas of the prostate as opposed to adenocarcinoma of the prostate, in which epithelial histology is predominant.

**S7. Comparison of EMT/MET biomarkers in DT, AT, CS-99, and DU145 cells. A.** QPCR of EMT master transcription factors in all model cell lines used in the present work. **B.** Western blotting of E-cadherin and vimentin in the model cell lines. GAPDH was included as a loading control.

**S8. Characterization of CS-99 human carcinosarcoma primary tumors and metastases. A.** CS-99 Rint+/Gint- and E-cadDipIIIcI^2^/Gint clones grow at equal rates. Statistically significant differences are indicated by asterisks. B. Hematoxylin staining of CS-99 primary tumors and metastases. Three representative images of primary tumors and metastases are shown. **C.** CS-99 primary tumors are negative for E-cadherin and positive for vimentin and Zeb1. Hoechst staining is indicated on the left of each image (blue) **D.** CS-99 Rint+/Gint- metastases (red) are negative for E-cadherin and express vimentin and Zeb1. Hoechst staining is indicated on the left of each image (blue). Note the positive E-cadherin staining of the surrounding lung tissue.
